# All-Cause and Cause-Specific Mortality Among Individuals With Hypochondriasis

**DOI:** 10.1001/jamapsychiatry.2023.4744

**Published:** 2023-12-13

**Authors:** David Mataix-Cols, Kayoko Isomura, Anna Sidorchuk, Daniel Rautio, Volen Z. Ivanov, Christian Rück, Susanna Österman, Paul Lichtenstein, Henrik Larsson, Ralf Kuja-Halkola, Zheng Chang, Isabell Brickell, Erik Hedman-Lagerlöf, Lorena Fernández de la Cruz

**Affiliations:** 1 Department of Clinical Neuroscience, Centre for Psychiatry Research, Karolinska Institutet and Stockholm Health Care Services, Region Stockholm, Stockholm, Sweden; 2 Department of Clinical Sciences, Lund University, Lund, Sweden; 3 Department of Medical Epidemiology and Biostatistics, Karolinska Institutet, Stockholm, Sweden; 4 School of Medical Sciences, Örebro University, Örebro, Sweden; 5 Department of Global Public Health and Primary Care, University of Bergen, Bergen, Norway; 6 Department of Biomedicine, Aarhus University, Aarhus, Denmark; 7 Division of Psychology, Department of Clinical Neuroscience, Karolinska Institutet, Stockholm, Sweden

## Abstract

**Importance:**

Hypochondriasis, also known as health anxiety disorder, is a prevalent, yet underdiagnosed psychiatric disorder characterized by persistent preoccupation about having serious and progressive physical disorders. The risk of mortality among individuals with hypochondriasis is unknown.

**Objective:**

To investigate all-cause and cause-specific mortality among a large cohort of individuals with hypochondriasis.

**Design, Setting, and Participants:**

This Swedish nationwide matched-cohort study included 4129 individuals with a validated *International Statistical Classification of Diseases and Related Health Problems, Tenth Revision* (*ICD-10*) diagnosis of hypochondriasis assigned between January 1, 1997, and December 31, 2020, and 41 290 demographically matched individuals without hypochondriasis. Individuals with diagnoses of dysmorphophobia (body dysmorphic disorder) assigned during the same period were excluded from the cohort. Statistical analyses were conducted between May 5 and September 27, 2023.

**Exposure:**

Validated *ICD-10* diagnoses of hypochondriasis in the National Patient Register.

**Main Outcome and Measures:**

All-cause and cause-specific mortality in the Cause of Death Register. Covariates included birth year, sex, county of residence, country of birth (Sweden vs abroad), latest recorded education, civil status, family income, and lifetime psychiatric comorbidities. Stratified Cox proportional hazards regression models were used to estimate the hazard ratios (HRs) and 95% CIs of all-cause and cause-specific mortality.

**Results:**

Of the 4129 individuals with hypochondriasis (2342 women [56.7%]; median age at first diagnosis, 34.5 years [IQR, 26.3-46.1 years]) and 41 290 demographically matched individuals without hypochondriasis (23 420 women [56.7%]; median age at matching, 34.5 years [IQR, 26.4-46.2 years]) in the study, 268 individuals with hypochondriasis and 1761 individuals without hypochondriasis died during the study period, corresponding to crude mortality rates of 8.5 and 5.5 per 1000 person-years, respectively. In models adjusted for sociodemographic variables, an increased rate of all-cause mortality was observed among individuals with hypochondriasis compared with individuals without hypochondriasis (HR, 1.69; 95% CI, 1.47-1.93). An increased rate was observed for both natural (HR, 1.60; 95% CI, 1.38-1.85) and unnatural (HR, 2.43; 95% CI, 1.61-3.68) causes of death. Most deaths from unnatural causes were attributed to suicide (HR, 4.14; 95% CI, 2.44-7.03). The results were generally robust to additional adjustment for lifetime psychiatric disorders.

**Conclusions and Relevance:**

This cohort study suggests that individuals with hypochondriasis have an increased risk of death from both natural and unnatural causes, particularly suicide, compared with individuals from the general population without hypochondriasis. Improved detection and access to evidence-based care should be prioritized.

## Key Points

**Question:**

Are individuals with hypochondriasis at increased risk of death due to natural and unnatural causes?

**Findings:**

In this Swedish nationwide matched-cohort study of 4129 individuals with a diagnosis of hypochondriasis and 41 290 demographically matched individuals without hypochondriasis, those with hypochondriasis had an increased risk of death from both natural and unnatural causes, particularly suicide.

**Meaning:**

This study suggests that individuals with hypochondriasis have an increased risk of mortality, mainly from potentially preventable causes.

## Teaser

This Swedish nationwide cohort study investigates all-cause and cause-specific mortality among a large cohort of individuals with hypochondriasis and a demographically matched cohort of individuals without hypochondriasis.

## Introduction

Hypochondriasis, also known as health anxiety disorder, is a prevalent psychiatric disorder^1^ characterized by persistent preoccupation about having 1 or more serious and progressive physical disorders.^2^ The preoccupation is accompanied by hypervigilance and a catastrophic interpretation of bodily signs, resulting in repetitive and excessive checking and reassurance-seeking behavior or maladaptive avoidance. The symptoms are clearly disproportionate and cause significant distress and impairment.^3^ Hypochondriasis is thought to be severely underdiagnosed due to the condition not being properly recognized or taken seriously by health professionals as well as to the negative connotations associated with the diagnostic label.^4,5^ However, as a symptom, health anxiety is highly prevalent in health care settings^6^ and is associated with a substantial use of health care resources.^1,4^ Hypochondriasis is widely regarded as a chronic disorder, with a low probability of remission without specialized treatment.^7,8^

Individuals with hypochondriasis have high rates of medical consultations, typically leading to a chain of laboratory and other tests, which are often unnecessary from a medical perspective and conceptualized as counterproductive from a psychological viewpoint.^9^ Theoretically, this high degree of vigilance may lead to the early detection and timely management of serious health conditions, potentially reducing mortality. However, there are several reasons to believe that this may not be the case. First, some individuals with hypochondriasis experience such high levels of health anxiety that they actually avoid contact with medical services altogether, risking the oversight of potentially serious illnesses.^7,10,11^ Second, chronic anxiety and depression, which are characteristic of the disorder, are known to be associated with a range of adverse health consequences, such as cardiovascular disorders and premature mortality.^12,13,14^ A longitudinal Norwegian cohort study of 7052 individuals found that those with self-reported symptoms of health anxiety were 73% more likely to develop ischemic heart disease after a 12-year follow-up compared with individuals with low health anxiety scores.^15^ Finally, suicide has not been formally investigated among individuals with hypochondriasis, but it could contribute to increased mortality among this group. To our knowledge, no studies have examined the risk of all-cause and cause-specific mortality among individuals with a clinical diagnosis of hypochondriasis.

This nationwide matched-cohort study linked several Swedish registers to investigate all-cause and cause-specific mortality among a large cohort of individuals with hypochondriasis. Unlike its international version, the Swedish version of the *International Statistical Classification of Diseases and Related Health Problems, Tenth Revision* (*ICD-10*) includes separate diagnostic codes for hypochondriasis and dysmorphophobia (body dysmorphic disorder),^16^ resulting in a unique resource for nationwide cohort studies.

## Methods

The Swedish Ethical Review Authority approved this study without requiring informed consent from participants because the study was register based and the included individuals were not identifiable at any time. This study followed the Strengthening the Reporting of Observational Studies in Epidemiology (STROBE) reporting guideline.

### Data Sources

We linked several Swedish population-based registers using the unique national identification numbers assigned to Swedish citizens.^17^ Sociodemographic data were extracted from the Census register, containing data from 1960; the Swedish Total Population Register, containing data on all Swedish inhabitants since 1968^18^; and the Longitudinal Integration Database for Health Insurance and Labour Market Studies, which, since 1990, annually integrates data on the labor market, education, and social sectors from all individuals living in Sweden.^19^ Dates of immigration into and emigration out of Sweden were extracted from the Migration Register.^18^ The National Patient Register includes diagnostic information on individuals admitted to a Swedish hospital since 1969, with complete data coverage for psychiatric disorders from 1973. From 2001, the National Patient Register also contains data on outpatient consultations in specialized care.^20^ Diagnoses are based on the *International Classification of Diseases, Eighth Revision* (1969-1986), *International Classification of Diseases, Ninth Revision* (1987-1996), and *ICD-10* (1997-onward) classification systems. The Cause of Death Register contains a record of all deaths in Sweden since 1952, with compulsory reporting nationwide.^21^ Each record contains the date of death and *ICD* codes for underlying and contributory causes of death. The Cause of Death Register covers more than 99% of all deaths among Swedish residents, including those occurring abroad, resulting in minimal loss of information.

### Matched Cohort

A matched-cohort design was used to estimate the risk of all-cause and cause-specific mortality among individuals with a diagnosis of hypochondriasis compared with individuals from the general population without hypochondriasis. The study population included individuals who lived in Sweden at any time between 1997 and 2020 at the age of 6 years or older. Individuals who died or emigrated prior to 1997 or their sixth birthday, whichever occurred last, and individuals who were born after December 31, 2014 (ie, <6 years before the study end on December 31, 2020), were excluded.

Exposed individuals were defined as those who received a diagnosis of hypochondriasis between January 1, 1997, and December 31, 2020, at the age of 6 years or older. They entered the cohort on the date of their first registered hypochondriasis diagnosis. Each exposed individual was matched on sex, birth year, and county of residence at the time of the first hypochondriasis diagnosis with 10 individuals from the study population who lived in Sweden but had not received a diagnosis of hypochondriasis by the date when the exposed individual received such diagnosis (ie, index date). At the time of matching, we further excluded all individuals who had emigrated and then returned to Sweden between their sixth birthday or 1997, whichever came later, and the index date to allow for a more complete register coverage. Individuals with and without hypochondriasis were followed up from the index date until the date of the outcome (ie, death), emigration from Sweden, or the end of the study (ie, December 31, 2020), whichever occurred first. For individuals without hypochondriasis, the follow-up was additionally censored at the date they changed the exposure status (ie, if they received a diagnosis of hypochondriasis during the study period), if relevant.

### Exposure

Individuals with at least 1 inpatient or outpatient diagnosis of hypochondriasis according to the Swedish *ICD-10* (code F45.2) were identified from the National Patient Register between January 1, 1997 (introduction of the *ICD-10* in Sweden), and December 31, 2020 (end of the study period). In line with previous register-based studies in related disorders,^22,23^ a lower age limit of 6 years was used to minimize the risk of misdiagnoses. The validity and reliability of the hypochondriasis *ICD* code in the National Patient Register are acceptable for register-based research (80% true positives; interrater reliability, 95%).^16^ As per previous validation work, we excluded individuals with a lifetime diagnosis of dysmorphophobia (Swedish *ICD-10* code F45.2A) from the entire cohort to limit the risk of misdiagnosis due to the close proximity of these codes.^16^

### Outcomes

For all individuals who died during the study period, we extracted all-cause mortality data and the specific underlying cause of death from the Cause of Death Register. We analyzed specific causes of death if the number of deaths in the group of exposed individuals was larger than 10. Specific causes of death with 10 or fewer individuals and causes of death classified under the “Codes for Special Purposes” chapter were grouped together under an “other causes of death” category. Causes of death were further grouped into natural and unnatural (ie, “external causes of morbidity and mortality”). From the group of unnatural causes, we also extracted the specific deaths due to suicide. See eTable 1 in Supplement 1 for the *ICD* codes.

### Covariates

Country of birth (Sweden or abroad) was extracted from the Swedish Total Population Register. Other sociodemographic variables were retrieved from the Census register or the Longitudinal Integration Database for Health Insurance and Labour Market Studies, using records corresponding to the end of the follow-up (ie, latest registered). These included the highest level of education (elementary education, ≤9 years; secondary education, 10-12 years; and higher education, >12 years), civil status (single, married or cohabiting, and divorced or widowed), and family income (lowest 20%, middle 60%, and top 20%).

Lifetime records of other psychiatric disorders were obtained from the National Patient Register and grouped into (1) neurodevelopmental disorders, (2) psychotic disorders, (3) bipolar disorders, (4) depressive disorders, (5) anxiety-related disorders, (6) eating disorders, and (7) substance use disorders (*ICD* codes in eTable 2 in Supplement 1). A lifetime approach to comorbidity was chosen because the time of recorded diagnosis in the registers often does not correspond to the date of disorder onset. Data on race and ethnicity are not collected as part of the Swedish registers and were therefore not available.

### Statistical Analysis

Statistical analyses were conducted between May 5 and September 27, 2023. Mortality rates per 1000 person-years for individuals with and without hypochondriasis were calculated. Survival curves by exposure status were calculated using Kaplan-Meier survival estimates. Stratified Cox proportional hazards regression analyses were used to estimate hazard ratios (HRs) with 95% CIs for mortality among individuals with a diagnosis of hypochondriasis compared with matched individuals without hypochondriasis, with time in years since the start of the follow-up as the underlying time scale. The analysis was first conducted for all causes of mortality and then separately for each specific cause (ie, individuals who died of other causes than the cause of interest were censored at the time of death). The initial model (model 1) adjusted for the matching variables, including birth year, sex, and county of residence at the index date. A second model (model 2) further adjusted for socioeconomic variables: country of birth and latest recorded educational level, civil status, and income. Missing data were coded as unknown and included in the models as nominal variables. Models 1 and 2 were repeated stratifying by sex. Additional models were used to assess whether further adjustment for lifetime psychiatric comorbidities (1 separate model for each comorbidity group) influenced the magnitude of the associations between hypochondriasis and mortality.

Data management and analyses were performed using SAS, version 9.4 (SAS Institute Inc). All tests used 2-tailed significance set at *P* < .05.

## Results

### Cohort Description

We identified 13 534 945 individuals living in Sweden between January 1, 1997, and December 31, 2020. After applying several exclusion criteria (eFigure in Supplement 1), we matched 4129 individuals with a diagnosis of hypochondriasis (2342 women [56.7%]; median age at first diagnosis, 34.5 years [IQR, 26.3-46.1 years]) with 41 290 individuals without hypochondriasis (23 420 women [56.7%]; median age at matching, 34.5 years [IQR, 26.4-46.2 years]) on sex, birth year, and county of residence at the time of diagnosis (Table 1).

**Table 1.** Sociodemographic and Clinical Characteristics of Study Participants

Variable	Individuals with hypochondriasis (n = 4129)	Matched individuals without hypochondriasis (n = 41 290)	χ^2^ or *t* Value	*P* value
Sex, No. (%)				
Female	2342 (56.7)	23 420 (56.7)	NA	NA
Male	1787 (43.3)	17 870 (43.3)		
Place of birth, No. (%)				
Sweden	3568 (86.4)	32 576 (78.9)	130.54^a^	<.001
Abroad	561 (13.6)	8714 (21.1)		
Educational level, No. (%)				
Elementary education	703 (17.0)	5706 (13.8)	54.31^a^	<.001
Secondary education	1553 (37.6)	16 696 (40.4)		
Higher education	1838 (44.5)	18 153 (44.0)		
Unknown	35 (0.9)	735 (1.8)		
Civil status, No. (%)				
Single	2576 (62.4)	22 665 (54.9)	104.75^a^	<.001
Married or cohabiting	1269 (30.7)	16 032 (38.8)		
Divorced or widowed	272 (6.6)	2472 (6.0)		
Unknown	12 (0.3)	121 (0.3)		
Family income level, No. (%)				
Lowest 20%	1326 (32.1)	8925 (21.6)	244.60^a^	<.001
Middle 60%	2081 (50.4)	24 173 (58.5)		
Top 20%	710 (17.2)	7884 (19.1)		
Unknown	12 (0.3)	308 (0.8)		
Any lifetime psychiatric comorbidities, No. (%)	3537 (85.7)	8213 (19.9)	8467.07^a^	<.001
Neurodevelopmental disorders	707 (17.1)	1757 (4.3)	1211.331^a^	<.001
Psychotic disorders	376 (9.1)	472 (1.1)	1299.13^a^	<.001
Bipolar disorders	319 (7.7)	599 (1.5)	746.38^a^	<.001
Depressive disorders	1998 (48.4)	3580 (8.7)	5496.88^a^	<.001
Anxiety-related disorders	3235 (78.4)	4960 (12.0)	11 169.91^a^	<.001
Eating disorders	332 (8.0)	687 (1.7)	695.96^a^	<.001
Substance use disorders	629 (15.2)	2220 (5.4)	620.34^a^	<.001
Follow-up time, mean (SD), y	7.7 (5.6)	7.7 (5.7)	0.83^b^	.41

Abbreviation: NA, not applicable.

^a^ χ^2^ Value.

^b^ *t* Value.

The demographic and clinical characteristics and mean follow-up times of the study cohorts are reported in Table 1. Individuals with hypochondriasis were significantly more likely than those without hypochondriasis to be born in Sweden, to be less educated, to be single, and to have a lower family income. Most individuals with hypochondriasis (3537 [85.7%]) had received at least 1 other lifetime psychiatric diagnosis, primarily anxiety-related and depressive disorders, compared with 8213 individuals in the group without hypochondriasis (19.9%; *P* < .001). These proportions were substantially lower (2222 individuals with hypochondriasis [53.8%] vs 5027 individuals without hypochondriasis [12.2%]; *P* < .001) if comorbidities were limited to those preceding the first diagnosis of hypochondriasis (eTable 3 in Supplement 1).

### All-Cause and Cause-Specific Mortality

**Figure 1.**
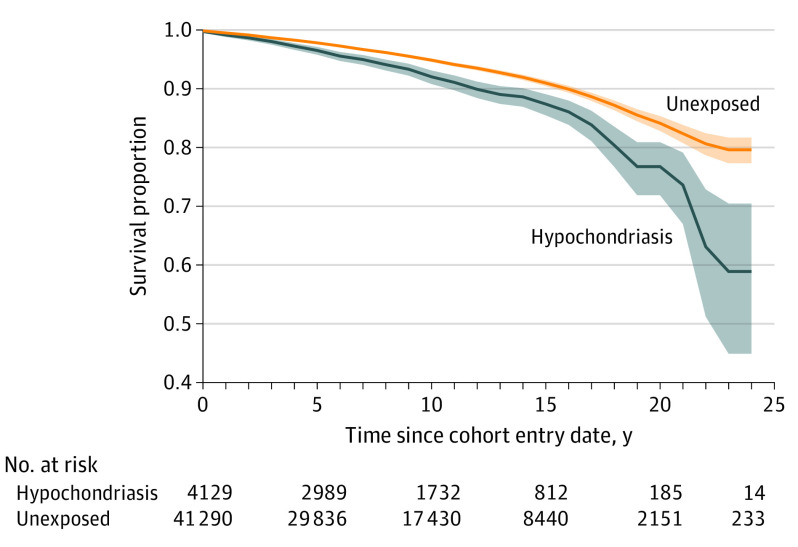
Survival Curves by Exposure Group The shaded areas indicate 95% CIs.

A total of 268 individuals with hypochondriasis and 1761 individuals without hypochondriasis died of all causes during the follow-up (crude mortality rates, 8.5 and 5.5 per 1000 person-years, respectively). Individuals with hypochondriasis died at an earlier mean (SD) age than the individuals without hypochondriasis (70.0 [16.8] years vs 75.1 [15.8]; *t* = 4.81; *P* < .001). Figure 1 displays the survival curves by exposure status.

**Figure 2.**
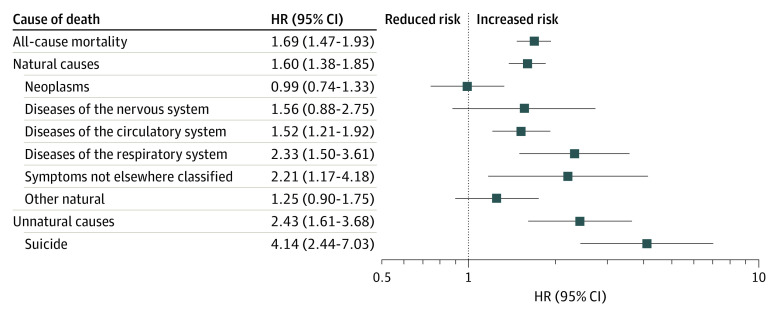
Hazard Ratios (HRs) for All-Cause and Cause-Specific Mortality Among Individuals With Hypochondriasis vs Matched Individuals Without Hypochondriasis Models adjusted for birth year, sex, county of residence, country of birth, latest recorded education, civil status, and family income.

The minimally adjusted risk estimates (model 1) showed that individuals with hypochondriasis had an 84% higher risk of all-cause mortality during the follow-up compared with individuals without hypochondriasis (HR, 1.84; 95% CI, 1.60-2.10) (Table 2; Figure 2). This risk remained significant when adjusting for all sociodemographic variables in model 2 (HR, 1.69; 95% CI, 1.47-1.93). Model 2 also showed significantly higher risks for both natural (HR, 1.60; 95% CI 1.38-1.85) and unnatural causes of death (HR, 2.43; 95% CI, 1.61-3.68).

**Table 2.** Hazard Ratios for All-Cause and Cause-Specific Mortality Among Individuals With a Diagnosis of Hypochondriasis Compared With Matched Individuals Without Hypochondriasis

Cause of death	No. (%)		Hazard ratio (95% CI)	
	Individuals with hypochondriasis (n = 4129)	Matched individuals without hypochondriasis (n = 41 290)	Model 1 (minimally adjusted)^a^	Model 2 (additionally adjusted for socioeconomic variables)^b^
All-cause mortality	268 (6.5)	1761 (4.3)	1.84 (1.60-2.10)^c^	1.69 (1.47-1.93)^c^
Natural causes	232 (5.6)	1646 (4.0)	1.70 (1.48-1.96)^c^	1.60 (1.38-1.85)^c^
Neoplasms	52 (1.3)	496 (1.2)	1.03 (0.77-1.37)	0.99 (0.74-1.33)
Diseases of the nervous system	14 (0.3)	88 (0.2)	1.59 (0.90-2.79)	1.56 (0.88-2.75)
Diseases of the circulatory system	87 (2.1)	598 (1.5)	1.60 (1.27-2.02)^c^	1.52 (1.21-1.92)^c^
Diseases of the respiratory system	26 (0.6)	116 (0.3)	2.42 (1.58-3.72)^c^	2.33 (1.50-3.61)^c^
Symptoms, signs, and abnormal clinical and laboratory findings not elsewhere classified	13 (0.3)	53 (0.1)	2.46 (1.34-4.52)^c^	2.21 (1.17-4.18)^c^
Other causes^d^	40 (1.0)	295 (0.7)	1.34 (0.97-1.87)	1.25 (0.90-1.75)
Unnatural causes	36 (0.9)	115 (0.3)	3.18 (2.18-4.63)^c^	2.43 (1.61-3.68)^c^
Suicide	29 (0.7)	53 (0.1)	5.57 (3.53-8.79)^c^	4.14 (2.44-7.03)^c^

^a^ Adjusted for all matching variables (ie, sex, birth year, county of residence at the time of hypochondriasis diagnosis) and country of birth (Sweden vs abroad).

^b^ Adjusted for all variables in model 1 and additionally for the highest level of education, family income level, and civil status.

^c^ Significant estimate.

^d^ Includes all groups of causes with 10 or fewer deaths and the causes of death classified in the *International Statistical Classification of Diseases and Related Health Problems, Tenth Revision* as “codes for special purposes.”

Regarding specific natural causes of death, the general pattern was one of increased risk for all causes except neoplasms. Most deaths from unnatural causes were attributed to suicide, with a 4-fold increased risk (HR, 4.14; 95% CI, 2.44-7.03) in model 2 (Table 2; Figure 2). The risk of all-cause and cause-specific mortality was generally similar among women and men with hypochondriasis (eTables 4 and 5 in Supplement 1).

In a post hoc analysis including a subcohort of 4006 individuals diagnosed from 2001 (when outpatient diagnoses were included in the register), we estimated, separately, the risks of death among individuals who first received a diagnosis of hypochondriasis in inpatient (n = 316) and outpatient settings (n = 3690). The risks were significantly higher in the inpatient group vs the outpatient group for all-cause mortality and for natural causes, but not for unnatural causes of death (eTable 6 in Supplement 1).

### The Role of Psychiatric Comorbidities

Analyses further controlling for various groups of lifetime psychiatric comorbidities showed that the risks of all-cause mortality and death from any natural causes remained statistically significant (Table 3). The risk of death from suicide was no longer statistically significant after adjustment for depressive and anxiety-related disorders. In a post hoc analysis limiting comorbidities to those recorded before the first diagnosis of hypochondriasis (and the corresponding date for individuals without hypochondriasis), the overall estimates were broadly unchanged, with suicide risk being attenuated but remaining statistically significant (eTable 7 in Supplement 1).

**Table 3.** Hazard Ratios for All-Cause and Cause-Specific Mortality Among Individuals With Hypochondriasis Compared With Matched Individuals Without Hypochondriasis, Further Adjusted for Different Groups of Lifetime Psychiatric Comorbidities

Cause of death	Hazard ratio (95% CI) for model 2^a^						
	Additionally adjusted for neurodevelopmental disorders	Additionally adjusted for psychotic disorders	Additionally adjusted for bipolar disorders	Additionally adjusted for depressive disorders	Additionally adjusted for anxiety-related disorders	Additionally adjusted for eating disorders	Additionally adjusted for substance use disorders
All-cause mortality	1.64 (1.42-1.88)^b^	1.45 (1.25-1.68)^b^	1.61 (1.40-1.85)^b^	1.23 (1.05-1.44)^b^	1.34 (1.12-1.59)^b^	1.63 (1.42-1.87)^b^	1.39 (1.20-1.60)^b^
Natural causes	1.58 (1.37-1.83)^b^	1.40 (1.20-1.63)^b^	1.54 (1.33-1.78)^b^	1.28 (1.09-1.51)^b^	1.37 (1.14-1.65)^b^	1.55 (1.34-1.80)^b^	1.37 (1.18-1.59)^b^
Neoplasms	1.00 (0.75-1.34)	0.99 (0.73-1.34)	0.98 (0.73-1.32)	0.94 (0.68-1.30)	0.91 (0.64-1.29)	0.97 (0.72-1.30)	0.95 (0.71-1.28)
Diseases of the nervous system	1.52 (0.86-2.72)	1.37 (0.74-2.54)	1.49 (0.84-2.66)	0.95 (0.49-1.83)	1.55 (0.71-3.38)	1.56 (0.87-2.77)	1.45 (0.81-2.58)
Diseases of the circulatory system	1.51 (1.20-1.91)^b^	1.37 (1.07-1.75)^b^	1.49 (1.18-1.89)^b^	1.37 (1.05-1.79)^b^	1.15 (0.86-1.55)	1.48 (1.17-1.88)^b^	1.41 (1.11-1.79)^b^
Diseases of the respiratory system	2.28 (1.47-3.55)^b^	2.02 (1.27-3.21)^b^	2.27 (1.46-3.52)^b^	1.90 (1.14-3.18)^b^	1.85 (1.05-3.24)^b^	2.26 (1.45-3.52)^b^	2.03 (1.29-3.19)^b^
Symptoms, signs, and abnormal clinical and laboratory findings not elsewhere classified	2.39 (1.26-4.52)^b^	2.09 (1.08-4.04)^b^	2.21 (1.17-4.19)^b^	1.33 (0.64-2.75)	1.37 (0.63-2.98)	2.16 (1.14-4.09)^b^	1.70 (0.86-3.37)
Other causes of death^c^	1.24 (0.89-1.74)	0.94 (0.65-1.37)	1.17 (0.83-1.65)	0.93 (0.63-1.37)	1.09 (0.71-1.67)	1.24 (0.88-1.74)	1.01 (0.71-1.44)
Unnatural causes	2.20 (1.44-3.36)^b^	1.95 (1.25-3.05)^b^	2.14 (1.39-3.29)^b^	1.13 (0.70-1.84)	1.21 (0.74-1.97)	2.32 (1.52-3.55)^b^	2.08 (1.31-3.29)^b^
Suicide	3.98 (2.32-6.82)^b^	3.44 (1.95-6.07)^b^	1.61 (1.40-1.85)^b^	1.37 (0.71-2.64)	1.86 (1.00-3.44)	3.85 (2.24-6.62)^b^	3.60 (1.98-6.55)^b^

^a^ Model 2 adjusts for all matching variables (ie, sex, birth year, county of residence at the time of hypochondriasis diagnosis), country of birth (Sweden vs abroad), and highest level of education, family income level, and civil status.

^b^ Significant estimate.

^c^ Includes all groups of causes with 10 or fewer deaths and the causes of death classified in the *International Statistical Classification of Diseases and Related Health Problems, Tenth Revision* as “codes for special purposes.”

## Discussion

To our knowledge, this was the first study to examine the causes of death among individuals with clinically diagnosed hypochondriasis. Several key findings emerged. First, individuals with a diagnosis of hypochondriasis had a significantly higher mortality rate than their counterparts without hypochondriasis and an 84% higher risk of all-cause mortality compared with individuals from the general population. The risks were broadly comparable for women and men with the disorder. Risks remained largely unchanged (with slightly attenuated estimates) after adjusting for socioeconomic variables known to be associated with life expectancy.^24^ The increased risk of death was already apparent early in the follow-up (Figure 1).

Second, individuals with hypochondriasis had increased risk of death due to both natural and unnatural causes compared with individuals without hypochondriasis. Among natural causes of death, the most common were circulatory system diseases, respiratory diseases, and “symptoms, signs and abnormal clinical and laboratory findings not elsewhere classified” (mainly including individuals whose death was due to unknown reasons). Our study could not address the mechanisms behind the findings. Multiple factors, probably acting in tandem, are likely to be associated with the increased risks. The avoidance of medical consultations that has been described for some individuals with severe hypochondriasis^7,10,11^ seems a less plausible explanation, given the observation that the risk of death from neoplasms was comparable in the groups with and without hypochondriasis. Other possible explanations appear more plausible, such as chronic stress leading to dysregulated hypothalamic-pituitary-adrenal axis function, immune dysfunction, chronic inflammation,^25,26,27,28^ lifestyle factors (eg, alcohol and substance use), the underrecognition of hypochondriasis as a genuine psychiatric disorder that requires treatment, and/or limited access to evidence-based treatment.

Third, individuals with a diagnosis of hypochondriasis had a more than 4-fold higher risk of death by suicide compared with individuals from the general population. To our knowledge, the risk of suicide in this group had not been previously quantified. A systematic review concluded that suicide attempts may be less frequent among individuals with hypochondriasis than among individuals without, although the included studies had methodological limitations.^29^ Clinicians should be aware that individuals with hypochondriasis are at risk of death by suicide, particularly if they have a lifetime history of depression and anxiety.

Fourth, the risks of death from all causes and natural causes were higher among individuals who first received a diagnosis in inpatient settings compared with individuals who first received a diagnosis in outpatient settings, suggesting that patients with more severe or complex symptoms requiring hospitalization are more likely to die. The risk of death from unnatural causes was not significantly different between groups, potentially due to limited statistical power.

Fifth, systematic adjustment for lifetime psychiatric disorders attenuated the magnitude of the risks, but, overall, these remained statistically significant. Suicide risk was no longer statistically significant after adjustment for depressive and anxiety-related disorders. However, these findings should be interpreted with caution given the high proportion of exposed individuals with these lifetime comorbidities and the resulting power issues. In a post hoc analysis limiting comorbidities to those recorded before hypochondriasis, suicide risk was attenuated but remained statistically significant. Even if the finding of increased mortality were not entirely specific to hypochondriasis, it is clear that hypochondriasis is not associated with protection from death.

Taken together, these findings illustrate a paradox, whereby individuals with hypochondriasis have an increased risk of death despite their pervasive fears of illness and death. In this study, most deaths could be classified as potentially preventable. Dismissing these individuals’ somatic symptoms as imaginary may have dire consequences. More should be done to reduce stigma and improve detection, diagnosis, and appropriate integrated (ie, psychiatric and somatic) care for these individuals.^30^ Evidence-based psychological treatments for hypochondriasis exist^31^ and, in some countries, are even available as low-threshold, guided self-help via the internet,^32,33^ substantially increasing access to treatment. The hope is that increased detection and access to evidence-based treatment will reduce somatic morbidity, suicidality, and mortality in this group.

### Strengths and Limitations

This study has some strengths; it was possible due to a unique quirk of the Swedish *ICD-10*, which has a separate code for hypochondriasis.^16^ Other strengths are the nationwide cohort design with a mean follow-up time of nearly 8 years and the adjustment for important sociodemographic variables and lifetime psychiatric comorbidities. Furthermore, to our knowledge, this cohort was probably the largest cohort of individuals with a formal diagnosis of hypochondriasis ever studied.

This study also has some limitations. First, hypochondriasis is underdiagnosed in Sweden, with only approximately 4000 cases registered in specialist services in 2 decades. Some reasons for this low number include that hypochondriasis symptoms are often wrongly considered secondary to other psychiatric disorders (eg, depression or anxiety), individuals are not taken seriously by health professionals, and the fact that the diagnosis carries stigma.^4,7^ These reasons mean that many individuals with this disorder are likely to be in the cohort without hypochondriasis, thus diluting the estimates; the true excess mortality among individuals with hypochondriasis may be even higher than reported. Second, we did not have data from primary care, where most individuals with hypochondriasis present for consultation. However, in Sweden, hypochondriasis is rarely diagnosed in primary care. Third, we controlled for sociodemographic variables at the end of follow-up, when they are most relevant for the outcome of interest. We acknowledge that this choice has some limitations, such as the difficulty of knowing if the covariate is a confounder or a mediator. However, we also reported a minimally adjusted model that did not include sociodemographic variables with broadly comparable results. Fourth, we could not adjust for somatization disorder because the code was not included in our database. However, our validation study showed that the risk of misdiagnosis is small.^16^ Fifth, while it is common practice in register studies to include undetermined causes of death as suicides,^34,35,36,37^ it is not possible to know if all were indeed suicides. Sixth, limited statistical power meant that we had to group several specific causes of death, and we could not conduct a co-sibling analysis, which would have helped rule out potential familial confounding.

## Conclusions

This cohort study is the first, to our knowledge, to suggest that individuals with hypochondriasis have an increased risk of all-cause mortality. The excess mortality was attributed to both natural and unnatural causes, particularly suicide, which can be generally classed as preventable.

## Notes

Supplement 1. **eFigure.** Flow Chart of Study Participants and Cohort Selection **eTable 1.** *International Classification of Diseases* (*ICD*) Codes Used to Group Specific Causes of Death in the Study **eTable 2.** Groups of Psychiatric Comorbidities and their Corresponding *International Classification of Diseases* (*ICD*) Codes **eTable 3.** Psychiatric Comorbidities Restricted to Those Recorded Before the First Diagnosis of Hypochondriasis or Index Date for the Matched Unexposed Individuals **eTable 4.** Hazard Ratios (HRs) With 95% Confidence Intervals (CIs) for All-Cause and Cause-Specific Mortality Among Women With Hypochondriasis, Compared to Their Matched Unexposed Individuals **eTable 5.** Hazard Ratios (HRs) With 95% Confidence Intervals (CIs) for All-Cause and Cause-Specific Mortality Among Men With Hypochondriasis, Compared to Their Matched Unexposed Individuals **eTable 6.** Results Statified According to Whether the Participants Were First Diagnosed With Hypochondriasis in Inpatient vs Outpatient Settings in a Subcohort From 2001 (n=4,006 Exposed and 40,060 Matched Unexposed Individuals) **eTable 7.** Hazard Ratios (HRs) With 95% Confidence Intervals (CIs) for All-Cause and Cause-Specific Mortality Among Individuals With Hypochondriasis, Compared to Their Matched Unexposed Individuals, Further Adjusted for Different Groups of Psychiatric Comorbidities (Restricted to Comorbidities Recorded Before the First Diagnosis of Hypochondriasis or Index Date for the Matched Unexposed Individuals)

Supplement 2.
Data Sharing Statement
