# Supplementary material for: All-Cause and Cause-Specific Mortality Among Individuals With Hypochondriasis
Source: JAMA Psychiatry. 2023 Dec 13;81(3):284–91. doi: 10.1001/jamapsychiatry.2023.4744 (PMC10719832; doi:10.1001/jamapsychiatry.2023.4744)
Supplement: Supplement 1. — eFigure. Flow Chart of Study Participants and Cohort Selection eTable 1. International Classification of Diseases (ICD) Codes Used to Group Specific Causes of Death in the Study eTable 2. Groups of Psychiatric Comorbidities and their Corresponding International Classification of Diseases (ICD) Codes eTable 3. Psychiatric Comorbidities Restricted to Those Recorded Before the First Diagnosis of Hypochondriasis or Index Date for the Matched Unexposed Individuals eTable 4. Hazard Ratios (HRs) With 95% Confidence Intervals (CIs) for All-Cause and Cause-Specific Mortality Among Women With Hypochondriasis, Compared to Their Matched Unexposed Individuals eTable 5. Hazard Ratios (HRs) With 95% Confidence Intervals (CIs) for All-Cause and Cause-Specific Mortality Among Men With Hypochondriasis, Compared to Their Matched Unexposed Individuals eTable 6. Results Statified According to Whether the Participants Were First Diagnosed With Hypochondriasis in Inpatient vs Outpatient Settings in a Subcohort From 2001 (n=4,006 Exposed and 40,060 Matched Unexposed Individuals) eTable 7. Hazard Ratios (HRs) With 95% Confidence Intervals (CIs) for All-Cause and Cause-Specific Mortality Among Individuals With Hypochondriasis, Compared to Their Matched Unexposed Individuals, Further Adjusted for Different Groups of Psychiatric Comorbidities (Restricted to Comorbidities Recorded Before the First Diagnosis of Hypochondriasis or Index Date for the Matched Unexposed Individuals) [file jamapsychiatry-e234744-s001.pdf]

Supplementary Online Content

Mataix-Cols D, Isomura K, Sidorchuk A, et al. All-cause and cause-specific mortality among individuals with hypochondriasis. *JAMA Psychiatry.* Published online December 13, 2023. doi:10.1001/jamapsychiatry.2023.4744

eFigure. Flow Chart of Study Participants and Cohort Selection
eTable 1. *International Classification of Diseases* (*ICD*) Codes Used to Group Specific Causes of Death in the Study
eTable 2. Groups of Psychiatric Comorbidities and their Corresponding *International Classification of Diseases* (*ICD*) Codes
eTable 3. Psychiatric Comorbidities Restricted to Those Recorded Before the First Diagnosis of Hypochondriasis or Index Date for the Matched Unexposed Individuals
eTable 4. Hazard Ratios (HRs) With 95% Confidence Intervals (CIs) for All-Cause and Cause-Specific Mortality Among Women With Hypochondriasis, Compared to Their Matched Unexposed Individuals
eTable 5. Hazard Ratios (HRs) With 95% Confidence Intervals (CIs) for All-Cause and Cause-Specific Mortality Among Men With Hypochondriasis, Compared to Their Matched Unexposed Individuals
eTable 6. Results Statified According to Whether the Participants Were First Diagnosed With Hypochondriasis in Inpatient vs Outpatient Settings in a Subcohort From 2001 (n=4,006 Exposed and 40,060 Matched Unexposed Individuals)
eTable 7. Hazard Ratios (HRs) With 95% Confidence Intervals (CIs) for All-Cause and Cause-Specific Mortality Among Individuals With Hypochondriasis, Compared to Their Matched Unexposed Individuals, Further Adjusted for Different Groups of Psychiatric Comorbidities (Restricted to Comorbidities Recorded Before the First Diagnosis of Hypochondriasis or Index Date for the Matched Unexposed Individuals)

This supplementary material has been provided by the authors to give readers additional information about their work.

© 2023 Mataix-Cols D et al. *JAMA Psychiatry.*

eFigure. Flow Chart of Study Participants and Cohort Selection

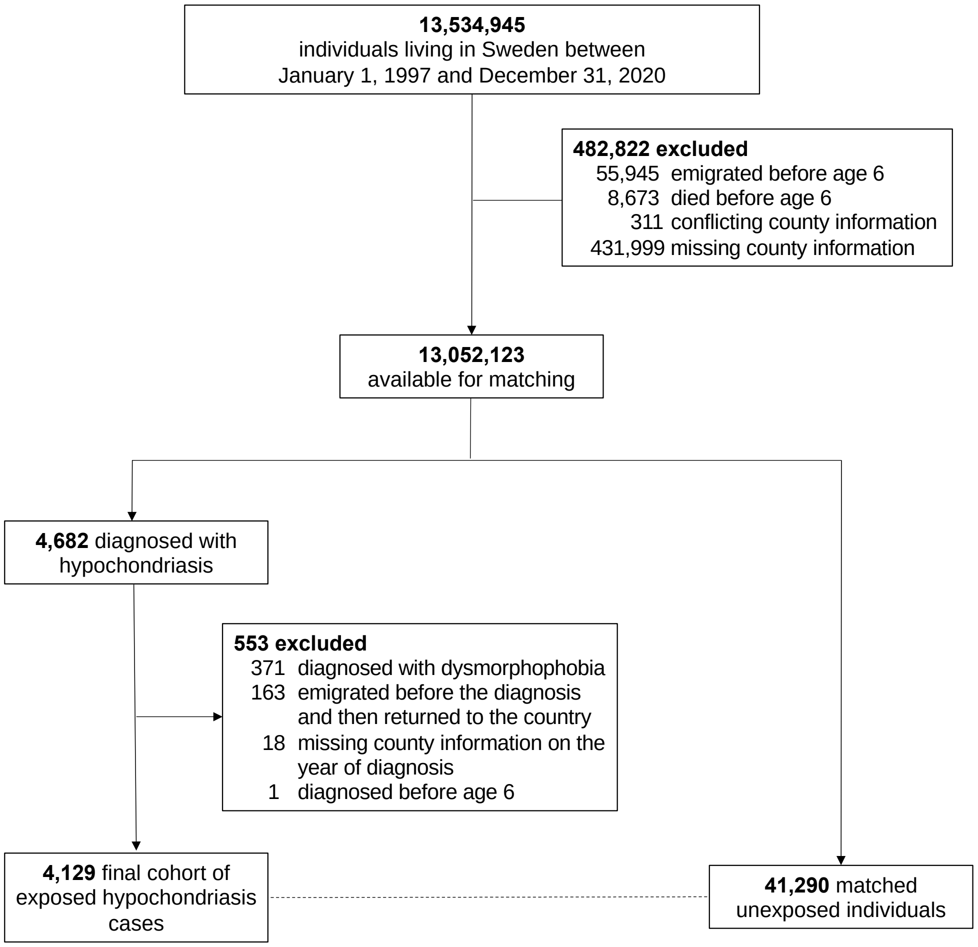

© 2023 Mataix-Cols D et al. *JAMA Psychiatry*.

eTable 1. *International Classification of Diseases* (*ICD*) Codes Used to Group Specific Causes of Death in the Study

Specific cause of death	Swedish ICD-10 diagnostic codes
Certain infectious and parasitic diseases[a]	A00−B99
Neoplasms	C00−D48
Diseases of the blood and blood-forming organs and certain disorders involving the immune mechanism[a]	D50−D89
Endocrine, nutritional, and metabolic diseases[a]	E00−E90
Mental and behavioral disorders[a]	F00−F99
Diseases of the nervous system	G00−G99
Diseases of the eye and adnexa[a]	H00−H59
Diseases of the ear and mastoid process[a]	H60−H95
Diseases of the circulatory system	I00−I99
Diseases of the respiratory system	J00−J99
Diseases of the digestive system[a]	K00−K93
Diseases of the skin and subcutaneous tissue[a]	L00−L99
Diseases of the musculoskeletal system and connective tissue[a]	M00−M99
Diseases of the genitourinary system[a]	N00−N99
Pregnancy, childbirth, and the puerperium[a]	O00−O99
Certain conditions originating in the perinatal period[a]	P00−P96
Congenital malformations, deformations and chromosomal abnormalities[a]	Q00−Q99
Symptoms, signs and abnormal clinical and laboratory findings, not elsewhere classified	R00−R99
Codes for special purposes[a]	U00-U99
External causes of morbidity and mortality	V01−Y98<br>**Suicide**:<br>X60-X84, Y10-34[b]

[a]Grouped together under 'Other causes of death' given the small number of deaths due to this cause in the study. [b] Our definition of death by suicide included both certain and undetermined causes to avoid underestimation of suicidal behavior rates and be consistent with suicide research (see e.g., Huguet et al. Arch Suicide Res. 2015;2:190-201; Neeleman et al Psychol Med .1997;27(2): 467-72; Sahlin et al. JAMA Psychiatry. 2017;74(6):615-621; Song et al., Am J Psychiatry. 2017;174(8):795-802).

© 2023 Mataix-Cols D et al. *JAMA Psychiatry*.

eTable 2. Groups of Psychiatric Comorbidities and their Corresponding *International Classification of Diseases* (*ICD*) Codes

Comorbidity groups	Corresponding Swedish ICD-8 diagnostic codes	Corresponding Swedish ICD-9 diagnostic codes	Corresponding Swedish ICD-10 diagnostic codes
**Neurodevelopmental disorders** (autism spectrum disorders, attention-deficit/ hyperactivity disorder[a], and Tourette syndrome and chronic tic disorder[b])	306.2	299, 307C, 314	F84, F90, F95
**Psychotic disorders** (schizophrenia and other psychotic disorders)	295 (minus 295.5), 297, 298 (minus 298.09)	295 (minus 295F), 297, 298 (minus 298A)	F20, F21, F22, F23, F24, F25 (minus F25.0), F28, F29
**Bipolar disorders**	296 (minus 296.00 and 296.20	296 (minus 296B)	F25.0, F30, F31, F34.0
**Depressive disorders** (major depressive disorder, persistent mood disorder, and unspecified mood disorder)	296.0, 296.2, 298.09	296B, 298A, 300E, 311	F32, F33, F34 (minus F34.0), F38, F39
**Anxiety-related disorders** (phobic, anxiety, obsessive-compulsive, reaction to severe stress, and adjustment disorders)	300.0, 300.2, 300.3, 307, 308.4	300A, 300C, 300D, 308, 309	F40.0, F40.1, F40.2, F41.0, F41.1, F42, F43
**Eating disorders**	–	307B, 307F	F50.0-F50.3, F50.9
**Substance use disorders**	303, 304	303, 304, 305A, 305X	F10-F16 and F18-19

[a]Individuals with attention-deficit/hyperactivity disorder (ADHD) were also identified by prescription of ADHD drugs, collected from the Prescription Drug Register, specifically Amfetamine (Anatomical Therapeutic Chemical [ATC] Classification System code: N06BA01), Dexamfetamine (N06BA02), Methylphenidate (N06BA04), Atomoxetine (N06BA09), and Lisdexamfetamine (N06BA12). [b]Tourette syndrome and chronic tic disorder were identified following the algorithm described in Rück et al. Validity and reliability of chronic tic disorders and obsessive-compulsive disorder diagnosis in the Swedish national Patient Register. BMJ Open. 2015;5(6):e007520.

© 2023 Mataix-Cols D et al. *JAMA Psychiatry*.

eTable 3. Psychiatric Comorbidities Restricted to Those Recorded Before the First Diagnosis of Hypochondriasis or Index Date for the Matched Unexposed Individuals

Variable	Individuals with hypochondriasis (N=4,129)	Matched unexposed individuals (N=41,290)	Chi-square test	p-value
**Any psychiatric comorbidity**, n (%)	2,222 (53.81)	5,027 (12.17)	4852.22	<.0001
Neurodevelopmental disorders	359 (8.69)	959 (2.32)	540.90	<.0001
Psychotic disorders	197 (4.77)	302 (0.73)	563.75	<.0001
Bipolar disorders	187 (4.53)	353 (0.85)	431.29	<.0001
Depressive disorders	1,140 (27.61)	2,140 (5.18)	2817.74	<.0001
Anxiety-related disorders	1,499 (36.30)	1,941 (4.70)	5355.52	<.0001
Eating disorders	215 (5.21)	467 (1.13)	421.65	<.0001
Substance use disorders	387 (9.37)	1,527 (3.70)	299.43	<.0001

© 2023 Mataix-Cols D et al. *JAMA Psychiatry*.

eTable 4. Hazard Ratios (HRs) With 95% Confidence Intervals (CIs) for All-Cause and Cause-Specific Mortality Among Women With Hypochondriasis, Compared to Their Matched Unexposed Individuals

Causes of death	Women with hypochondriasis (N=2,342) n (%)	Matched unexposed women (N=23,420) n (%)	HR (95% CI) Model 1 (minimally adjusted)[a]	HR (95% CI) Model 2 (additionally adjusted for socioeconomic variables)[b]
**All-cause mortality**	139 (5.94)	943 (4.03)	**1.82 (1.51-2.19)**	**1.69 (1.40-2.05)**
**Natural causes of death**	124 (5.29)	907 (3.87)	**1.67 (1.37-2.03)**	**1.58 (1.29-1.92)**
Neoplasms	36 (1.54)	289 (1.23)	1.21 (0.86-1.72)	1.15 (0.81-1.63)
Diseases of the circulatory system	45 (1.92)	306 (1.31)	**1.78 (1.29-2.45)**	**1.70 (1.22-2.35)**
Diseases of the respiratory system	15 (0.64)	60 (0.26)	**2.70 (1.53-4.78)**	**2.58 (1.43-4.66)**
Other causes of death[c]	28 (1.20)	252 (1.08)	1.10 (0.74-1.62)	1.04 (0.70-1.54)
**Unnatural causes of death**	15 (0.64)	36 (0.15)	**4.21 (2.30-7.71)**	**3.22 (1.58-6.55)**
Suicides	13 (0.56)	16 (0.07)	**7.92 (3.81-16.5)**	**5.22 (2.15-12.7)**

Note: Significant estimates are highlighted in bold. [a]Adjusted for all matching variables (i.e., sex, birth year, county of residence at the time of hypochondriasis diagnosis) and country of birth (Sweden vs. abroad). [b]Adjusted for all variables in Model 1 and additionally for the latest recorded highest level of education, family income level, and civil status. [c]Includes all groups with a small number of deaths (≤10) in the cases cohort for at least one of the sexes and the causes of death classified in the ICD as ‘codes for special purposes’.

© 2023 Mataix-Cols D et al. *JAMA Psychiatry*.

eTable 5. Hazard Ratios (HRs) With 95% Confidence Intervals (CIs) for All-Cause and Cause-Specific Mortality Among Men With Hypochondriasis, Compared to Their Matched Unexposed Individuals

Causes of death	Men with hypochondriasis (N=1,787) n (%)	Matched unexposed men (N=17,870) n (%)	HR (95% CI) Model 1 (minimally adjusted)[a]	HR (95% CI) Model 2 (additionally adjusted for socioeconomic variables)[b]
**All-cause mortality**	129 (7.22)	818 (4.58)	**1.85 (1.53-2.25)**	**1.65 (1.35-2.01)**
**Natural causes of death**	108 (6.04)	739 (4.14)	**1.74 (1.41-2.15)**	**1.59 (1.28-1.97)**
Neoplasms	16 (0.90)	207 (1.16)	0.76 (0.46-1.26)	0.75 (0.45-1.26)
Diseases of the circulatory system	42 (2.35)	292 (1.63)	**1.45 (1.04-2.01)**	1.35 (0.96-1.88)
Diseases of the respiratory system	11 (0.62)	56 (0.31)	**2.12 (1.10-4.07)**	**2.00 (1.02-3.91)**
Other causes of death[c]	39 (2.18)	184 (1.03)	**2.19 (1.55-3.11)**	**2.03 (1.42-2.90)**
**Unnatural causes of death**	21 (1.18)	79 (0.44)	**2.71 (1.67-4.39)**	**2.12 (1.25-3.57)**
Suicides	16 (0.90)	37 (0.21)	**4.49 (2.49-8.12)**	**3.66 (1.84-7.26)**

Note: Significant estimates are highlighted in bold. [a]Adjusted for all matching variables (i.e., sex, birth year, county of residence at the time of hypochondriasis diagnosis) and country of birth (Sweden vs. abroad). [b]Adjusted for all variables in Model 1 and additionally for the latest recorded highest level of education, family income level, and civil status. [c]Includes all groups with a small number of deaths (≤10) in the cases cohort for at least one of the sexes and the causes of death classified in the ICD as ‘codes for special purposes’.

© 2023 Mataix-Cols D et al. *JAMA Psychiatry*.

eTable 6. Results Statified According to Whether the Participants Were First Diagnosed With Hypochondriasis in Inpatient vs Outpatient Settings in a Subcohort From 2001 (n=4,006 Exposed and 40,060 Matched Unexposed Individuals)

	Individuals with hypochondriasis	Matched unexposed individuals	HR (95% CI) Model 1 (minimally adjusted)[c]	HR (95% CI) Model 2 (additionally adjusted for socioeconomic variables)[d]	Chi-square[e]	p-value[e]
**Causes of death**	**n (%)**	**n (%)**				
**All-cause mortality**						
Inpatients[a]	72 (22.78)	369 (11.68)	**2.49 (1.90-3.25)**	**2.39 (1.82-3.14)**	12.68	0.0004
Outpatients[b]	126 (3.41)	945 (2.56)	**1.43 (1.18-1.72)**	**1.30 (1.07-1.58)**		
**Natural causes of death**						
Inpatients[a]	62 (19.62)	349 (11.04)	**2.27 (1.70-3.02)**	**2.25 (1.68-3.01)**	11.23	0.0008
Outpatients[b]	107 (2.90)	870 (2.36)	**1.32 (1.08-1.62)**	1.22 (0.99-1.50)		
**Unnatural causes of death**						
Inpatients[a]	10 (3.16)	20 (0.63)	**4.91 (2.30-10.5)**	**3.12 (1.26-7.73)**	0.35	0.5521
Outpatients[b]	19 (0.51)	75 (0.20)	**2.59 (1.56-4.29)**	**2.26 (1.30-3.94)**		

Note: Significant estimates are highlighted in bold. [a]Analyses are based on 316 exposed and 3,160 matched unexposed individuals. [b]Analyses are based on 3,690 exposed and 36,900 matched unexposed individuals. [c]Adjusted for all matching variables (i.e., sex, birth year, county of residence at the time of hypochondriasis diagnosis) and country of birth (Sweden vs. abroad). [d]Adjusted for all variables in Model 1 and additionally for the latest recorded highest level of education, family income level, and civil status. [e]Formal test of heterogeneity comparing the HR (95% CI) for the risk of death in inpatients vs outpatients with hypochondriasis (corresponding to Model 2).

© 2023 Mataix-Cols D et al. *JAMA Psychiatry*.

eTable 7. Hazard Ratios (HRs) With 95% Confidence Intervals (CIs) for All-Cause and Cause-Specific Mortality Among Individuals With Hypochondriasis, Compared to Their Matched Unexposed Individuals, Further Adjusted for Different Groups of Psychiatric Comorbidities (Restricted to Comorbidities Recorded Before the First Diagnosis of Hypochondriasis or Index Date for the Matched Unexposed Individuals)

Causes of death	HR (95% CI) Model 2, additionally adjusted for neurodevelopmental disorders[a]	HR (95% CI) Model 2, additionally adjusted for psychotic disorders[a]	HR (95% CI) Model 2, additionally adjusted for bipolar disorders[a]	HR (95% CI) Model 2, additionally adjusted for depressive disorders[a]	HR (95% CI) Model 2, additionally adjusted for anxiety-related disorders[a]	HR (95% CI) Model 2, additionally adjusted for eating disorders[a]	HR (95% CI) Model 2, additionally adjusted for substance use disorders[a]
**All-cause mortality**	**1.66 (1.45-1.91)**	**1.55 (1.35-1.79)**	**1.62 (1.41-1.86)**	**1.38 (1.18-1.60)**	**1.48 (1.27-1.73)**	**1.65 (1.44-1.90)**	**1.54 (1.33-1.77)**
**Natural causes of death**	**1.59 (1.37-1.84)**	**1.47 (1.27-1.71)**	**1.53 (1.32-1.78)**	**1.38 (1.17-1.62)**	**1.47 (1.25-1.72)**	**1.57 (1.36-1.82)**	**1.48 (1.27-1.71)**
Neoplasms	1.01 (0.76-1.35)	0.97 (0.73-1.31)	0.97 (0.73-1.31)	0.97 (0.71-1.33)	1.01 (0.74-1.38)	0.97 (0.72-1.29)	0.97 (0.72-1.29)
Diseases of the nervous system	1.56 (0.88-2.75)	1.47 (0.82-2.62)	1.54 (0.87-2.73)	1.23 (0.65-2.35)	1.39 (0.72-2.65)	1.60 (0.91-2.84)	1.55 (0.87-2.74)
Diseases of the circulatory system	**1.52 (1.20-1.92)**	**1.45 (1.14-1.84)**	**1.51 (1.20-1.91)**	**1.44 (1.11-1.87)**	**1.40 (1.09-1.80)**	**1.50 (1.18-1.89)**	**1.48 (1.17-1.88)**
Diseases of the respiratory system	**2.46 (1.59-3.82)**	**2.17 (1.38-3.41)**	**2.29 (1.48-3.57)**	**2.01 (1.22-3.30)**	**2.12 (1.29-3.51)**	**2.32 (1.49-3.59)**	**2.13 (1.36-3.35)**
Symptoms, signs and abnormal clinical and laboratory findings, not elsewhere classified	**2.32 (1.22-4.39)**	**2.31 (1.21-4.41)**	**2.19 (1.16-4.14)**	1.64 (0.78-3.45)	1.61 (0.79-3.28)	**2.02 (1.04-3.89)**	**2.06 (1.06-4.00)**
Other causes of death[b]	1.24 (0.89-1.74)	1.04 (0.72-1.49)	1.16 (0.82-1.64)	1.06 (0.73-1.55)	1.16 (0.81-1.67)	1.28 (0.91-1.79)	1.14 (0.81-1.62)
**Unnatural causes of death**	**2.28 (1.49-3.48)**	**2.24 (1.46-3.42)**	**2.32 (1.52-3.53)**	1.47 (0.90-2.41)	**1.66 (1.03-2.69)**	**2.37 (1.55-3.61)**	**2.66 (1.71-4.15)**
Suicide	**4.04 (2.35-6.95)**	**3.88 (2.25-6.70)**	**3.93 (2.29-6.77)**	**1.99 (1.03-3.86)**	**3.04 (1.70-5.44)**	**3.99 (2.33-6.83)**	**4.95 (2.80-8.77)**

Note: Significant estimates are highlighted in bold. [a]Model 2 adjusts for all matching variables (i.e., sex, birth year, county of residence at the time of hypochondriasis diagnosis), country of birth (Sweden vs. abroad), and highest level of education, family income level, and civil status. [b]Includes all chapters with (≤10) deaths and the causes of death classified in the ICD as 'codes for special purposes'.

© 2023 Mataix-Cols D et al. *JAMA Psychiatry*.
