# Supplementary material for: All-Cause and Cause-Specific Mortality Among Individuals With Hypochondriasis
Source: JAMA Psychiatry. 2023 Dec 13;81(3):284–91. doi: 10.1001/jamapsychiatry.2023.4744 (PMC10719832; doi:10.1001/jamapsychiatry.2023.4744)
Supplement: Supplement 2. — Data Sharing Statement [file jamapsychiatry-e234744-s002.pdf]

# Data Sharing Statement

Mataix-Cols. All-Cause and Cause-Specific Mortality Among Individuals With Hypochondriasis. *JAMA Psychiatry*. Published December 13, 2023. doi:10.1001/jamapsychiatry.2023.4744

**Data**
**Data available:** No

**Additional Information**
**Explanation for why data not available:** Nationwide register data cannot be made publicly available according to Swedish and European law.
